# Structural and Functional Alterations in the Contralesional Medial Temporal Lobe in Glioma Patients

**DOI:** 10.3389/fnins.2020.00010

**Published:** 2020-02-20

**Authors:** Taoyang Yuan, Zhentao Zuo, Jianyou Ying, Lu Jin, Jie Kang, Songbai Gui, Rui Wang, Chuzhong Li

**Affiliations:** ^1^Beijing Neurosurgical Institute, Capital Medical University, Beijing, China; ^2^State Key Laboratory of Brain and Cognitive Science, Institute of Biophysics, Chinese Academy of Sciences, Beijing, China; ^3^CAS Center for Excellence in Brain Science and Intelligence Technology, Chinese Academy of Sciences, Shanghai, China; ^4^Sino-Danish College, University of Chinese Academy of Sciences, Beijing, China; ^5^Department of Neurosurgery, Beijing Tiantan Hospital, Capital Medical University, Beijing, China; ^6^Beijing Institute for Brain Disorders Brain Tumour Center, Beijing, China

**Keywords:** glioma, medial temporal lobe, brain structure and function, structural magnetic resonance imaging analysis, functional connectivity

## Abstract

**Background:**

The human brain has an extraordinary ability to functionally change or reorganize its structure in response to disease. The aim of this study is to assess the structural and functional plasticity of contralesional medial temporal lobe (MTL) in patients with unilateral MTL glioma.

**Methods:**

Sixty-eight patients with unilateral MTL glioma (left MTL glioma, *n* = 33; right MTL glioma, *n* = 35) and 40 healthy controls were recruited and scanned with 3D T1 MRI and rest-fMRI. We explored the structure of the contralesional MTL using voxel-based morphometry (VBM) and assessed the memory networks of the contralesional hemisphere using resting-state functional connectivity (rs-FC). The association between FC and cognitive function was assessed with partial correlation analysis.

**Results:**

Compared with healthy controls, both patient groups exhibited (1) a large cluster of voxels with gray matter (GM) volume decrease in the contralesional MTL using region of interest (ROI)-based VBM analysis (cluster level *p* < 0.05, FDR corrected); and (2) decreased intrahemispheric FC between the posterior hippocampus (pHPC) and posterior cingulate cortex (PCC) (*p* < 0.01, Bonferroni corrected). Intrahemispheric FC between the pHPC and PCC was positively correlated with cognitive function in both patient groups.

**Conclusion:**

Using multi-modality brain imaging tools, we found structural and functional changes in the contralesional MTL in patients with unilateral MTL glioma. These findings suggest that the contralesional cortex may have decompensation of structure and function in patients with unilateral glioma, except for compensatory structural and functional adaptations. Our study provides additional insight into the neuroanatomical and functional network changes in the contralesional cortex in patients with glioma.

## Introduction

The human brain has an extraordinary ability to functionally change or reorganize its structure in response to internal and environmental changes, physiologic modifications, novel learning, cognitive demand, and behavioral experiences (Draganski and May, 2008; Raz and Lindenberger, 2013; Voelcker-Rehage and Niemann, 2013; Li et al., 2014). This property, known as neuroplasticity, has been demonstrated extensively in many brain regions by functional and structural magnetic resonance imaging (MRI) in the past two decades. In terms of brain lesions, including those resulting from brain injuries, strokes, and brain tumors, functional imaging studies have identified the brain to maintain its original features or to support functional recovery by recruiting other parts of the brain, such as the perilesional brain, contralesional brain, and homologous function of the brain, in order to compensate for lost functions (Small et al., 2002; Freundlieb et al., 2015; Fisicaro et al., 2016; Han et al., 2017, 2018).

The medial temporal lobe (MTL), consisting of the hippocampus and parahippocampal gyrus (PHG), plays a critical role in memory and cognition (Squire et al., 2004). Neuroimaging studies have reported structural and functional alterations in the bilateral mesial temporal lobe cortex that are associated with memory and cognitive deficits in patients with mesial temporal lobe epilepsy (mTLE) (Alessio et al., 2004; Bonilha et al., 2004). MTL resection for the treatment of medically intractable temporal lobe epilepsy is commonly associated with episodic memory impairment after surgery (Pillon et al., 1999; Savage et al., 2002). In patients with a normal range of memory capacity following unilateral MTL resection, task-based functional connectivity analysis has revealed functional reorganization in the contralesional hippocampus and medial prefrontal cortex (Jeong et al., 2019). Recently, many studies have demonstrated that the MTL forms two functionally distinct memory circuits, with each circuit delineating distinct anatomical and functional connections to other related brain regions (Kahn et al., 2008; Fanselow and Dong, 2010; Poppenk and Moscovitch, 2011; Libby et al., 2012). The anterior memory network is formed by the direct connection of the anterior hippocampus (aHPC) to the entorhinal cortex (EC), temporal pole (TP), and orbitofrontal cortex (OFC). The posterior hippocampus (pHPC) connects to the PHG, posterior cingulate cortex (PCC), thalamus (THA), dorsolateral prefrontal cortex (DLPFC), and lingual gyrus (LG) forming the posterior memory network. Interestingly, some recent studies investigated the anterior and posterior memory networks in patients with mTLE and found that FC alterations in the memory network were associated with memory deficit (Voets et al., 2014).

Gliomas are the most prevalent primary tumors of the brain and spinal cord, and their outstanding characteristics are infiltration and destruction of brain tissue leading to neurological dysfunction (Chen et al., 2017). Imaging studies have reported that gliomas that occur in “eloquent” areas, such as Broca’s area, Wernicke’s area, or the primary motor cortex, may not result in detectable functional deficits due to functional reorganization by recruiting homologous non-dominant, contralateral tissues and by recruiting and reshaping neuronal connections (Duffau et al., 2003; Kośla et al., 2012; Chivukula et al., 2018). More recently, a structural MRI study reported that slow-growing but massive lesion infiltration of the insula induced a significant increase in gray matter (GM) volume in the contralesional insula (Almairac et al., 2018). To date, no study has reported whether structural and functional plasticity of contralesional MTL exists in patients with unilateral MTL glioma.

In this study, to ascertain this possible structural and functional reorganization, the structure of the contralesional MTL and the memory network of the contralesional hemisphere were investigated in 68 patients with unilateral MTL glioma using voxel-based morphometry (VBM) and resting-state functional connectivity (rs-FC) analysis.

## Materials and Methods

### Participants

A total of 68 patients with unilateral MTL glioma who underwent therapy in the Department of Neurosurgery, Beijing Tiantan Hospital, were enrolled in our study. Among these subjects, 33 patients with left MTL glioma (left MTL group) and 35 patients with right MTL glioma (right MTL group) were included. The inclusion criteria for the patient group were 16–70 years of age and histologically proven glioma. No patients received steroids at the time of MRI. The exclusion criteria were as follows: a history of stroke, cerebral trauma, brain surgery or brain radiotherapy, other intracranial abnormalities (i.e., arachnoid cyst), glioma extended to both the left and right MTLs, an inability to complete the MRI examinations, or preprocessing issues (i.e., head motion).

The healthy controls consisted of 40 neurologically intact participants. The following individuals were excluded: those with a history of neurodegenerative, neurodevelopmental, or psychiatric diseases; those with substance use disorders for alcohol or heroin; those unable to complete the MRI examinations; or preprocessing issues (i.e., head motion).

This study was approved by the medical ethics committee of Beijing Tiantan Hospital. Written informed consent was obtained from the legal representatives of all the patients and all the healthy volunteers in accordance with the Declaration of Helsinki.

### Image Acquisition

All subjects were scanned on the 3.0-T Siemens scanner with a standard head coil. 3D T1-weighted sagittal image was acquired (192 slices, slice thickness/gap = 1/0.5 mm, repetition time = 2530 ms, echo time = 2.55 ms, acquisition matrix = 512 × 512; flip angle = 12°, FOV = 256 mm × 256 mm with an in-plane resolution of 0.7 mm × 0.7 mm). The rs-fMRI data were acquired with an echo-planar image sequence [30 axial slices, number of volumes = 200, slice thickness/gap = 5/0.5 mm, repetition time = 2000 ms, echo time = 30 ms, acquisition matrix = 64 × 64, field of view (FOV) = 192 mm × 192 mm with an in-plane resolution of 3.0 mm × 3.0 mm]. The T2 image parameters were as follows: repetition time = 5000 ms; echo time = 105 ms; flip angle = 150°; 33 slices; FOV = 199 mm × 220 mm; voxel size = 0.49 mm × 0.49 mm × 3.9 mm; and matrix = 406 × 448. During scanning, all the participants were instructed to relax, stare at a fixation point on the center of the screen, and avoid thinking about anything.

### Tumor Masking

**FIGURE 1:**
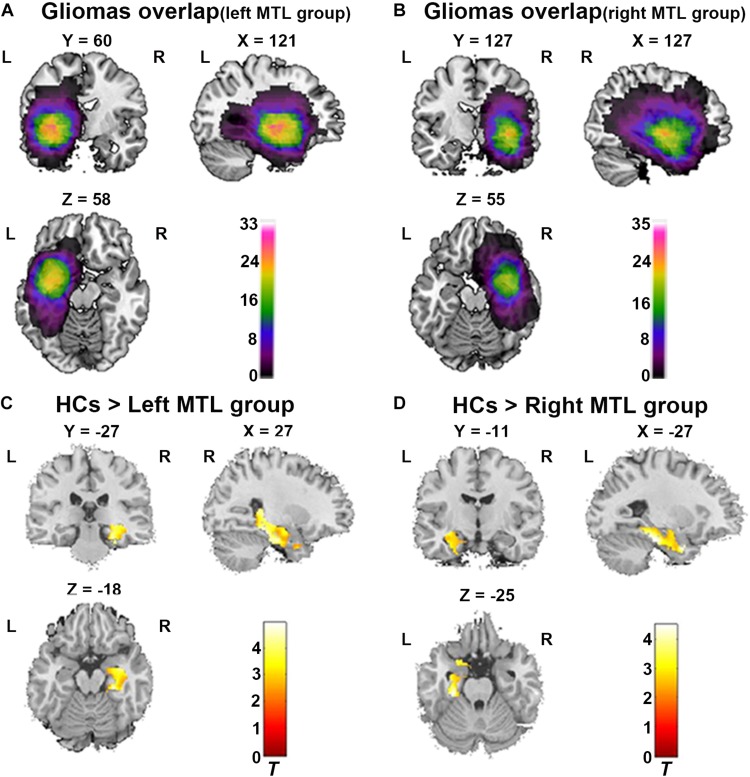
ROI-based VBM analysis between both patient group and healthy controls (HCs). **(A)** Glioma overlap map in the left MTL group. **(B)** Glioma overlap map in the right MTL group. **(C)** ROI-based VBM analysis showed significant decrease of GMV in contralesional MTL in the left MTL group compared to HCs (Cluster level *p* < 0.05, FDR corrected, cluster size > 50). **(D)** ROI-based VBM analysis showed significant decrease of GMV in contralesional MTL in the right MTL group compared to HCs (Cluster level *p* < 0.05, FDR corrected, cluster size > 50). L, Left hemisphere; R, right hemisphere.

To define the anatomical location of the tumor in each patient, T2 image for each patient was registered to the Montreal Neurological Institute (MNI) template using the standard non-linear spatial normalization algorithm provided by SPM12. In our study, the hyperintensities around tumor including perifocal edema were included in the tumor masks. The tumor mask was manually traced slice by slice by an experienced neurosurgeon using MRIcron software^1^; representative examples of glioma and segmentation are shown in Supplementary Figure 1. A volume of interest (VOI) was created and tumor volumes were generated with MRIcron for each patient. Finally, all tumor masks were overlapped in Ch2bet template. The tumor overlap maps are displayed in Figures 1A,B.

### Cognitive Data

The Mini-Mental Status Exam (MMSE) was tested in all patients and healthy controls.

### Data Preprocessing

Structural image preprocessing was conducted using SPM12^2^ and the CAT12 toolbox^3^ in the MATLAB environment (MATHWORKS, CA, United States). To summarize, structural images were manually reoriented and shifted to define the anterior commissure as the origin (mm coordinate 0, 0, 0). Then, using the module “Segment Data” of CAT12, 3D T1-weighted images were segmented with the segment module implemented in SPM12 into GM, white matter (WM), and cerebrospinal fluid (CSF). The modulated warped GM images were then normalized to MNI-152 standard space with an isotropic voxel resolution of 1.5 mm × 1.5 mm × 1.5 mm. Next, using “Display one slice for all images,” we checked the data quality to determine if the segmentation and normalization procedures yielded reasonable results. Using the “Estimate TIV” module, the TIV of all subjects were obtained. In addition, checked sample homogeneity was also performed to identify outliers by visualizing the correlation between the volumes. The modulated GM map of each individual was smoothed with an 8-mm full-width at half-maximum (FWHM) Gaussian kernel. Statistical analyses were performed on the smoothed images generated during the last preprocessing step.

The rs-fMRI data were preprocessed using statistical Parametric Mapping toolbox (SPM12^4^) and Data Processing & Analysis of Brain Imaging toolbox (DPABI^5^) (Yan et al., 2016). First, the first 10 volumes of the functional images were excluded to ensure steady baseline condition. The remaining 190 volumes were corrected for acquisition time delay between slices. Realignment was conducted to correct head motion. The participants with head motion >3 mm in maximum displacement or >3 mm rotation in angular motion was excluded from our study. Furthermore, in order to minimize the impact of micro-movements on volume changes, the mean frame displacement (FD) was calculated for each subject and the bad points were identified by a threshold of FD (>0.5 mm) as well as one-forward and two-back neighbors according to a previously published formula (Power et al., 2012). After the motion correction, the structural image was coregistered to the mean functional image and the transformed structural image was segmented into GM, WM, and CSF. Then, the segmented images were normalized to MNI space using Diffeomorphic Anatomical Registration Through Exponentiated Lie algebra (DARTEL) algorithm (Ashburner, 2007). Next, we normalized the motion-corrected functional volumes to the MNI space using the normalization parameters for their respective structure images (resampling voxel size = 3 mm × 3 mm × 3 mm). Nuisance covariates including 24 head motion parameters, WM signal, CSF signal, and linear trend were regressed out. Functional images were spatially smoothed with an 8-mm FWHM isotropic Gaussian kernel. Finally, temporal bandpass filtering (0.01–0.1 Hz) was performed to reduce low-frequency drift and high-frequency noise.

### VBM Analysis

Two-sample *t*-test was applied to assess GM volume (GMV) between the patient groups (left MTL or right MTL group) and healthy controls in SPM12 with TIV, age, and sex as covariates. In this part, proportional scaling was used for global normalization and an absolute masking with a threshold of 0.2 was selected referenced to previous studies (Ridgway et al., 2012; Almairac et al., 2018). Then, to ascertain possible structural reorganization in contralesional MTL, MTL region of interest (ROI) masks using the WFU-Pickatlas toolbox based on the Anatomical Automatic Labeling (AAL) template were applied to explore structural changes in MTL. Significance was determined using pFDR (false discovery rate) < 0.05 at the cluster level and cluster sizes *k* > 50.

### Rs-FC Analysis

After rs-fMRI data preprocessing, calculations of functional connectivity were carried out in DPABI. According to the previous study, the aHPC, EC, OFC, and TP were components of the anterior memory networks, while the pHPC, PHG, PCC, THA, DLPFC, and LG constituted the posterior memory networks. The spherical ROIs with a diameter of 4 mm in the aHPC (L: *x y z* = −18 −14 −18; R: *x y z* = 18 −14 −18), pHPC (L: *x y z* = −23 −26 −15; R: *x y z* = 25 −26 −15), EC (L: *x y z* = −26 −20 −30; R: *x y z* = 26 −20 −30), and PHG (L: *x y z* = −26 −40 −12; R: *x y z* = 26 −40 −12) were created using the coordinates of MNI space (Kahn et al., 2008; Damoiseaux et al., 2016). The ROIs of DLPFC, OFC, TP, PCC, THA, and LG were obtained from the WFU-Pickatlas toolbox based on the AAL template. To assess the validity of the networks extracted, we analyzed the average functional connectivity map of the right MTL tumor patients and HCs using the right aHPC and pHPC, respectively, as seeds to calculate their functional connectivity with the voxels in the ipsilateral hemisphere with a single-sample *t* -test (Cluster level *p* < 0.001, FDR corrected, cluster size > 50) in DPABI. Similarly, average functional connectivity map of the left MTL tumor patients and HCs were created. Next, functional connectivity measures were computed from aHPC to EC, OFC, and TP as well as from pHPC to PHG, PCC, THA, DLPFC, and LG in the contralesional hemisphere. We extracted the residual BOLD time course from these ROI and then estimated their first-level correlation map by calculating Pearson’s correlation coefficients between these ROIs. To increase normality and improve second-level general linear model analyses, correlation coefficients were transformed into Fisher’s “*Z*” scores. Then, we calculated ROI-to-ROI functional connectivity for each subject. To examine group differences, two-sample *t*-tests with Bonferroni correction (*p* < 0.05) were performed between the patient groups (left MTL or right MTL group) and the HCs. The memory networks were displayed with BrainNet Viewer (Xia et al., 2013).

### Statistical Analyses

Non-imaging data statistical analyses were conducted with SPSS version 25. One-way ANOVA was applied to compare the quantitative variables (age, MMSE) among three groups. Chi-square test was applied to compare the sex difference in three groups. The partial correlations were applied to confirm the relationship between the FC and MMSE by correcting for covariance (age, sex, and education). The significance level was set at a two-sided *p* < 0.05.

## Results

### Demographic and Clinical Factors

The detailed demographic and clinical characteristics of subjects are summarized in Tables 1, 2. All patients underwent surgical treatment, and the postoperative pathological diagnosis was glioma. No significant differences in age, sex, or educational level were found among the right MTL, left MTL, and HCs. The MMSE scores were significantly lower in the left and right MTL groups than in the HCs (*F* = 4.14, *p* = 0.019).

**TABLE 1:** Demographic and clinical characteristics among patient groups and healthy controls (HCs).

Characteristic	Left MTL group	Right MTL group	HCs	*P*-value
Numbers	33	35	40	NA
Age (mean ± SD) (years)	44.6 ± 10.1	43.4 ± 12.9	44.0 ± 11.9	0.905^a^
Sex ratio, F/M (*n*)	15/18	16/19	18/22	0.998^b^
Handedness, R/L (*n*)	33/0	35/0	40/0	NA
MMSE (mean ± SD)	27.9 ± 3.0	27.8 ± 2.8	29.3 ± 1.4	0.019^a^
Education (mean ± SD) (years)	11.2 ± 3.5	11.5 ± 4.1	13.0 ± 3.8	0.104^a^

*^*a*^One-way ANOVA, two-sided. ^*b*^Chi-square test, two-sided.*

**TABLE 2:** Histological class and tumor types.

Characteristic	Left MTL group	Right MTL group
**Histological grade**		
I	1	1
II	15	16
III	8	8
IV	9	10
**Tumor type**		
Oligodendroglioma	2	4
Astrocytoma	9	4
Oligoastrocytomas	4	8
Anaplastic oligodendroglioma	1	4
Anaplastic astrocytoma	4	4
Glioblastoma	9	10
Anaplastic oligoastrocytomas	3	0
Ganglioglioma	1	1

### ROI-Based VBM Analysis

#### GM Volume Alteration in Right MTL in Patients With Left MTL Glioma

In the left MTL group (*n* = 33), the map of individual glioma overlap is shown in Figure 1A. As shown in Figure 1C, significant GMV decreases were found in the left MTL group compared to the HCs in the contralesional MTL (Table 3, FDR corrected, cluster level *p* < 0.05).

**TABLE 3:** VBM analyses for patient groups compared with HCs in the contralesional MTL.

Analysis	Group	Cluster size (in voxels)	Peak MNI coordinate			Peak level *t* value
				
			*X*	*Y*	*Z*	
VBM	HCs vs. Left MTL group	2936	38	−34	−16	4.94
			26	−16	−27	4.86
			26	−30	−16	4.47
	HCs vs. Right MTL group	1480	−27	−27	−22	4.50
			−33	−9	−15	4.34
			−27	2	−32	4.21

**T*-values were determined by a two-sample *t*-test (Cluster level *p* < 0.05, FDR corrected), voxel size = 1.5 × 1.5 × 1.5 mm.*

#### GM Volume Alteration in Left MTL in Patients With Right MTL Glioma

In the right MTL group (*n* = 35), the map of individual glioma overlap is shown in Figure 1B. As shown in Figure 1D, significant GMV decreases were found in the right MTL group compared to the HCs in the contralesional MTL (Table 3, FDR corrected, cluster level *p* < 0.05).

### ROI-Based Resting-State FC Analysis in the Contralesional Hemispheric Memory Network

**FIGURE 2:**
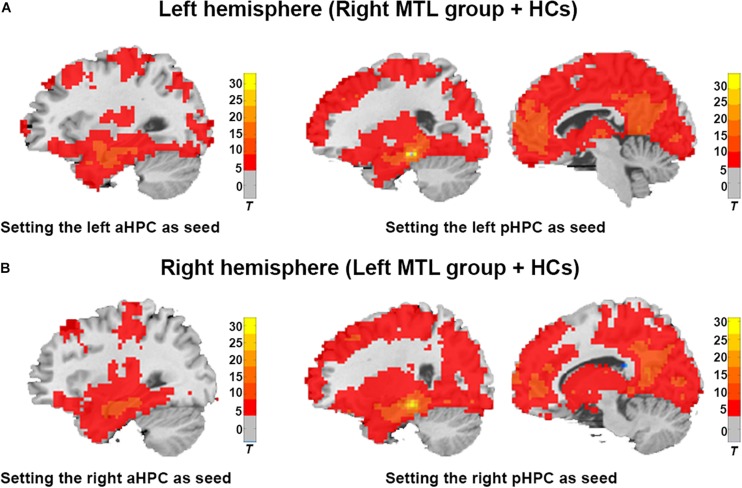
Illustration of the average brain functional connectivity map based on the aHPC and pHPC seeds in the ipsilateral hemisphere. **(A)** Average functional connectivity map of the right MTL group and HCs with a single-sample *t*-test (Cluster level *p* < 0.001, FDR corrected, cluster size > 50). **(B)** Average functional connectivity map of the left MTL group and HCs with a single-sample *t*-test (Cluster level *p* < 0.001, FDR corrected, cluster size > 50).

**FIGURE 3:**
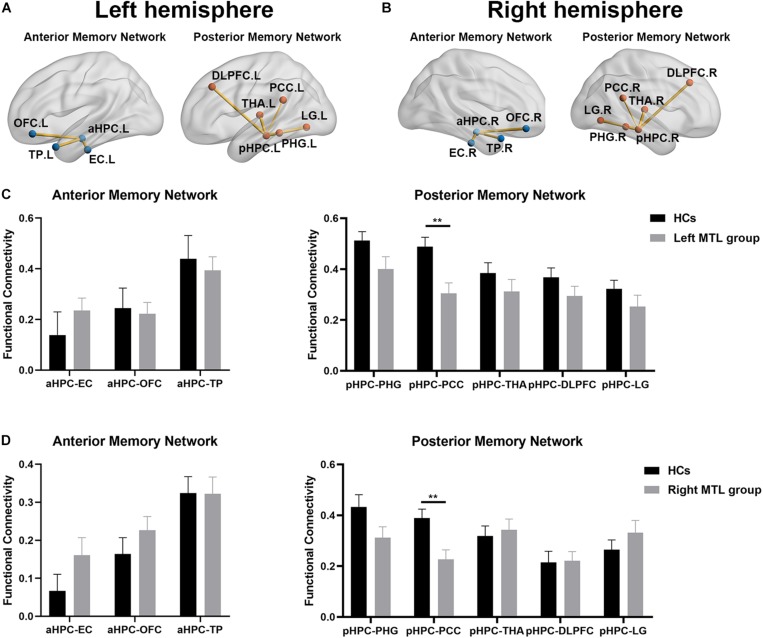
The alteration of memory network in both patient groups compared to HCs. The functional connectivity map of anterior memory network and posterior memory network in contralesional hemisphere in the right MTL group **(A)** and the left MTL group **(B)** compared to HCs. **(C)** Statistical comparison of functional connectivity values at anterior memory network and posterior memory network between HCs and the left MTL group in the contralesional hemisphere. Significant decrease of FC from the pHPC to the PCC in the left MTL group compared to HCs. Two-sample *t*-tests with Bonferroni correction (*p* < 0.05). **(D)** Statistical comparison of functional connectivity values at anterior memory network and posterior memory network between HCs and right MTL group in the contralesional hemisphere. Significant decrease of FC from the pHPC to the PCC in right MTL group compare to HCs. Two-sample *t*-tests with Bonferroni correction (*p* < 0.05). Double asterisks denote significant between-group differences (*p* < 0.01). HCs, healthy controls; aHPC, anterior hippocampus; pHPC, posterior hippocampus; EC, entorhinal cortex; TP, temporal pole; OFC, orbitofrontal cortex; PHG, parahippocampal gyrus; PCC, posterior cingulate cortex; THA, thalamus; DLPFC, dorsolateral prefrontal cortex; LG, lingual gyrus.

As shown in Figures 2A,B, the average functional connectivity map using the aHPC and pHPC as seeds proves the validity of our memory network extraction in tumor patients and healthy controls. We next investigated whether the strength of FC differed among regions forming anterior and posterior memory networks in the contralesional hemisphere. In the right MTL group, the result showed reduced FC between pHPC and PCC in the posterior memory network compared to HCs (Figures 3A,D, *p* < 0.01, Bonferroni corrected). Notably, in the left MTL group, reduced FC between pHPC and PCC in the posterior memory network compared to HCs was also found (Figures 3B,C, *p* < 0.01, Bonferroni corrected). In the two patient groups, there was no significant difference between aHPC and cortical regions in the anterior memory network compared to HCs.

### Relationship Between Resting-State FC and Cognitive Performance

**FIGURE 4:**
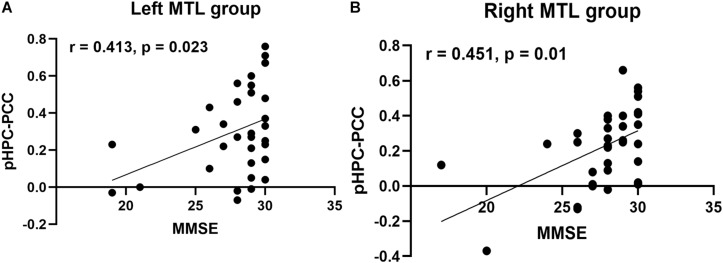
Correlation analysis between FC from pHPC to PCC and MMSE score in both patient groups. **(A)** Partial correlation analysis between FC from the pHPC to the PCC and MMSE score in the left MTL group, correcting age, sex, and education (*r* = 0.413, *p* = 0.023). **(B)** Partial correlation analysis between FC from the pHPC to the PCC and MMSE score in the right MTL group, correcting age, sex, and education (*r* = 0.451, *p* = 0.01).

Next, we tested whether an association existed between decreased FC and MMSE scores in both patient groups by partial correlation analysis corrected for age, sex, and education. In the left MTL group, the results showed a significant positive correlation between FC from the pHPC to PCC and the MMSE score (Figure 4A, *r* = 0.413, *p* < 0.023). Similarly, a significant positive correlation between FC from the pHPC to PCC and the MMSE score was also detected in the right MTL group (Figure 4B, *r* = 0.451, *p* < 0.01).

## Discussion

Neuroplasticity is the ability of the brain to reorganize itself during normal development and in response to illness; it has been extensively reported in normal adult humans, ischemic stroke patients, and brain injury patients (Kong et al., 2016). In glioma patients, increasing studies have demonstrated that both ipsilateral and contralateral brain regions are recruited to compensate for the neurological dysfunction caused by glioma-induced injury (Bryszewski et al., 2012; Krieg et al., 2013; Auriat et al., 2015; Fisicaro et al., 2016). To date, no study has detected whether the structural and functional plasticity of contralesional MTL in patients with unilateral MTL glioma exist. In the current study, we investigated the structural and functional plasticity of contralesional MTL in patients with unilateral MTL glioma using VBM and rs-FC. Our results showed that compared with healthy controls, both patient groups (left MTL group and right MTL group) exhibited a large cluster of voxels with GMV decrease in contralesional MTL and decreased intra-hemispheric FC from the pHPC to the PCC in the posterior memory network. In addition, intra-hemispheric FC from the pHPC to the PCC was positively correlated with cognitive function in both patient groups.

Voxel-based morphometry enables voxel-wise statistical comparison of local GMV between groups and is a most widely used automated technique for the analysis of structural brain images (Ashburner and Friston, 2000). In our study, both MTL groups revealed significant decrease of GMV in contralesional MTL compared with healthy controls. All patients in our study had been diagnosed with gliomas for the first time and no patients had undergone surgery, radiotherapy, or chemotherapy. The decrease of GMV in contralesional MTL indicated that unilateral glioma may cause structural atrophy of contralesional cortex, rather than induce structural compensatory plasticity. The decrease of MTL volume may be secondary to several potential processes such as tumor biology, functional decompensation, and inflammation (Ye and Sontheimer, 1999; Liu et al., 2012). However, the definite mechanism needs to be further investigated.

The atrophy of brain structure generally indicates the functional impairment. For example, in Alzheimer’s disease (AD), previous studies have demonstrated strong associations between memory function decline and MTL atrophy (Nho et al., 2012; La Joie et al., 2013). In the patients with brain tumor, some studies have suggested that radiation therapy can cause the atrophy of hippocampus, which was associated with learning and memory deficits (Tome et al., 2011). Recent evidence of functional dissociations among hippocampal memory systems from animal experiments and human fMRI studies have demonstrated that MTL and cortical regions constituted two main memory networks (anterior and posterior memory network), which are primarily associated with verbal memory and non-verbal memory (Kahn et al., 2008; Aggleton, 2011; Voets et al., 2014). The memory network provides a novel method to investigate the functional alteration of MTL and mechanisms affecting cognitive and memory decline in health and disease. In both patient groups compared to HCs, we found significantly decreased FC between pHPC and PCC in the contralesional hemisphere. This result also indicated that both patient groups exhibited symmetry change of FC between pHPC and PCC in disease. PCC is a critical node in default mode network (DMN) and flexibly integrates information from different functional networks (Leech et al., 2012). The alteration of functional connectivity starting from the PCC was closely related to memory and cognitive function, especially FC from PCC to hippocampus (McCormick et al., 2013). In the patient with epilepsy, previous studies have reported a selective vulnerability of FC between the hippocampus and posterior cingulate/precuneus (PCUN) regions of the DMN. In mTLE patients with hippocampal sclerosis, significant decrease of functional and structural connectivity between the PCC/PCUN and bilateral mesial temporal lobes were also reported (Liao et al., 2011). In addition, a previous study has reported that contralateral FC between pHPC and PCC was lowest in patients who were impaired on memory testing in patients with mTLE (Voets et al., 2014). In our study, only MMSE scores were collected and significant differences between patient groups and HCs were observed, especially the part about memory function. Thus, we tested whether the association between decreased FC and MMSE score existed in both patient groups by partial correlation analysis correcting for age, sex, and education. Relative to HCs, our observations showed significant positive correlation between contralateral FC of pHPC to PCC and MMSE score in both patient groups. However, the MMSE was designed as a quick-and-dirty test of general cognitive function and the decrease in score cannot directly reflect the memory impaired in patients with MTL glioma. This correlation cannot prove the relationship between memory network changes and memory function very well. In the future, much more appropriate neuropsychological test batteries should be applied to examine the memory function in patients with MTL glioma. Considering the occupying effect and invasion of gliomas, the alterations of inter-hemispheric FC of nodes within anterior and posterior memory networks were not investigated in the current study. Taken together, our results suggested no compensatory adaptive changes at contralesional memory network in the patients with unilateral MTL glioma.

In our study, the main limitation is lack of detail memory function test batteries, such as episodic memory testing or Word List and Logical Memory tasks from the Wechsler Memory Scale, to investigate memory impaired in patients with MTL glioma and their relationships with the alterations of structure and function in contralesional MTL. Besides, there was a lack of task-based fMRI data to study the alteration of neural activity in contralesional MTL and relationship between task performance and memory deficit.

## Conclusion

Using multiple-modality brain imaging tools, we found structural and functional alteration of the contralesional MTL in patients with unilateral MTL glioma. These findings suggest that the contralesional cortex may have decompensation of structure and function in patients with unilateral glioma, except compensatory structural and functional adaptation. Our study provided additional insight into neuroanatomical and functional network changes of contralesional cortex in patients with glioma.

## Data Availability Statement

The datasets generated for this study are available on request to the corresponding author.

## Ethics Statement

The studies involving human participants were reviewed and approved by medical ethics committee of Beijing Tiantan Hospital. The patients/participants provided their written informed consent to participate in this study.

## Author Contributions

CL and TY conceptualized and designed the study. TY, JY, LJ, JK, SG, and CL recruited the participants and completed the screening assessments. TY, ZZ, LJ, and RW analyzed the data and performed the statistical analysis. TY and CL wrote the first draft of the manuscript. All authors revised the manuscript and approved the final manuscript.

## Conflict of Interest

The authors declare that the research was conducted in the absence of any commercial or financial relationships that could be construed as a potential conflict of interest.

## Footnotes

1 https://www.mccauslandcenter.sc.edu/crnl/tools

2 http://www.fil.ion.ucl.ac.uk/spm

3 http://www.neuro.uni-jena.de/cat/

4 https://www.fil.ion.ucl.ac.uk/spm/software/spm12/

5 http://www.restfmri.net/
