# Supplementary material for: Structural and Functional Alterations in the Contralesional Medial Temporal Lobe in Glioma Patients
Source: Front Neurosci. 2020 Feb 20;14:10. doi: 10.3389/fnins.2020.00010 (PMC7044242; doi:10.3389/fnins.2020.00010)
Supplement: Supplementary file 1 [file Table_1.DOCX]

***Supplementary Material***


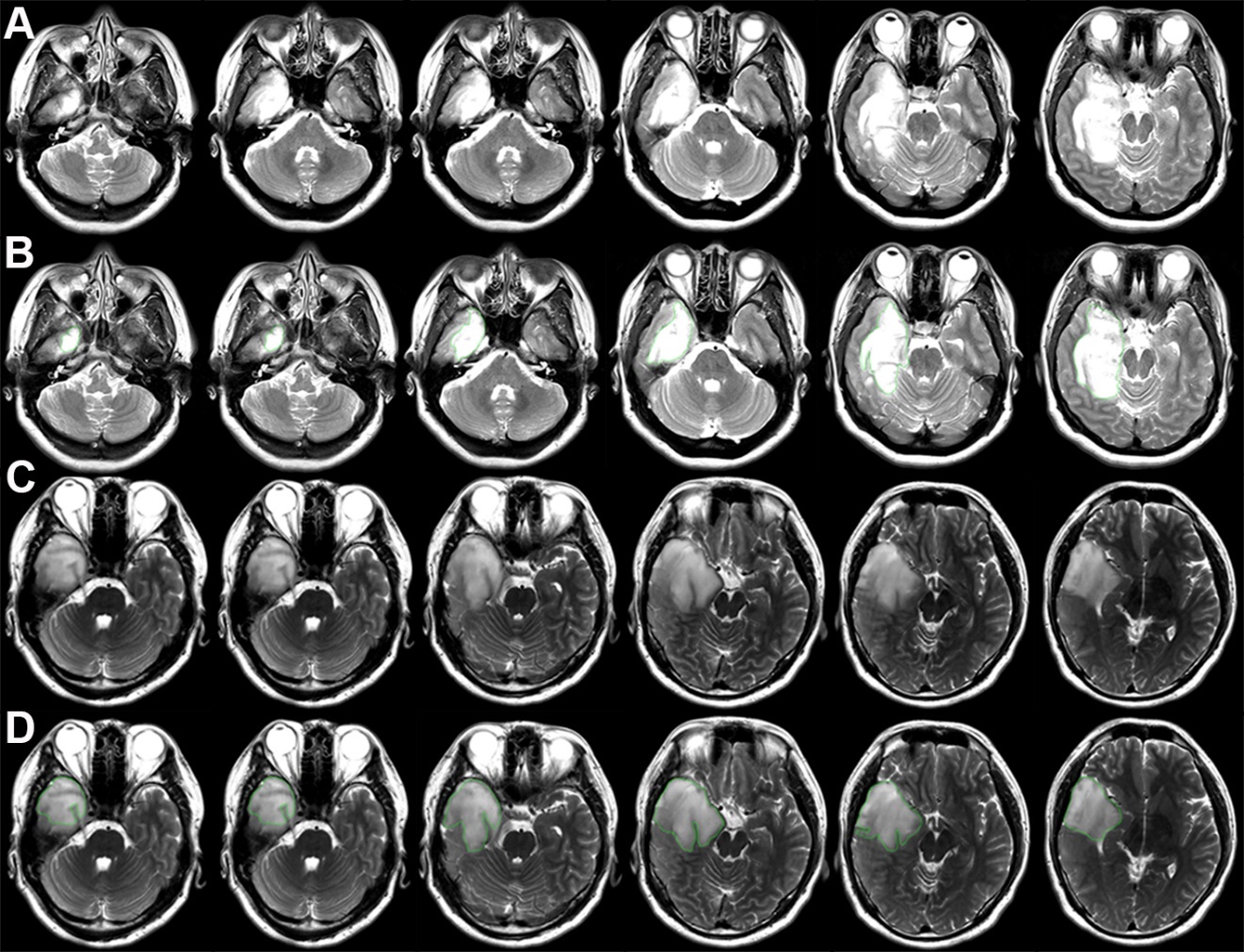


**Supplementary Figure 1. Two representative examples of glioma and tumor mask segmented.**

(A) primary T2 images and (B) tumor mask was traced sliced by sliced on T2 images in patient one. (C) Primary T2 images and (D) tumor mask was traced sliced by sliced on T2 images in patient two.
